# From acidity to sweetness: a comprehensive review of carbon accumulation in grape berries

**DOI:** 10.1186/s43897-024-00100-8

**Published:** 2024-06-05

**Authors:** Lizhen Lu, Serge Delrot, Zhenchang Liang

**Affiliations:** 1 State Key Laboratory of Plant Diversity and Prominent Crop, Beijing Key Laboratory of Grape Science and Oenology, Institute of Botany, Chinese Academy of Sciences, Beijing, 100093 China; 2 China National Botanical Garden, Beijing, 100093 China; 3 University of Chinese Academy of Sciences, Beijing, 100049 China; 4 Bordeaux University, Bordeaux Sciences Agro, INRAE, UMR EGFV, ISVV, Villenave d’Ornon, 33882 France

**Keywords:** Sugar accumulation, Sugar metabolism, Sugar transporter, Regulatory factors, Hexose, Acid metabolism

## Abstract

Most of the carbon found in fruits at harvest is imported by the phloem. Imported carbon provide the material needed for the accumulation of sugars, organic acids, secondary compounds, in addition to the material needed for the synthesis of cell walls. The accumulation of sugars during fruit development influences not only sweetness but also various parameters controlling fruit composition (fruit “quality”). The accumulation of organic acids and sugar in grape berry flesh cells is a key process for berry development and ripening. The present review presents an update of the research on grape berry development, anatomical structure, sugar and acid metabolism, sugar transporters, and regulatory factors.

## Introduction

The grapevine (Vitis vinifera L.), as a prominent fruit crop, is cultivated extensively around the world, with a cultivation history extending over 11,000 years (Dong et al. 2023). Grape berries serve a wide range of activities centered around table grapes, raisins, juice, wine and spirits, catering to a diverse array of markets (Kuhn et al. 2014; Li et al. 2021). As of 2022, the total global vineyard surface area was approximately 7.28 million hectares (https://www.statista.com/statistics/240635/total-vineyard-areas-worldwide-and-in-europe). The revenue in the fresh fruits market, which includes grapes, is expected to be around US$ 726 billion in 2024, with a forecasted annual growth of 6.58% (CAGR 2024–2028) (https://www.statista.com/outlook/cmo/food/fruits-nuts/fresh-fruits/worldwide). The global wine market, which is a major segment of grape consumption, was valued at USD 326 billion and is expected to grow at a CAGR of 4.4% during 2021–2026 (https://www.mordorintelligence.com/industry-reports/grapes-market). Additionally, red wine market specifically is expected to grow to $136 billion in 2028 at a CAGR of 5.2% (https://finance.yahoo.com/news/red-wine-global-market-report-161300922.html). The grapes market itself is expected to reach USD 215 billion in 2024 and grow at a CAGR of 7.10% to reach USD 303.20 billion by 2029 (https://www.mordorintelligence.com/industry-reports/grapes-market). These figures pinpoint the major economic impact of grape berry production and use.

During ripening, the berries accumulate high concentration of hexoses (1.1 M) in the vacuoles of flesh cells (Shahood et al. 2020; Du et al. 2023). The sweetness of grape berries impacts directly the sensory quality of berries and wine (Conde et al. 2007; Yang et al. 2023; Jiang et al. 2024). Sugar accumulation in the berries is a finely tuned outcome of numerous physiological processes including photosynthesis in the leaves, long-distance transport in phloem and unloading in sink organs (Martínez-Esteso et al. 2011; Lecourieux et al. 2014; Castellarin et al. 2016; Zhang et al. 2019; Martínez-Lüscher and Kurtural 2023). Till the eighties, in some wine areas, it was not uncommon to add sugars to the must (chaptalization) under controlled practices when the berry sugar content was not high enough to produce wines. Due to global climate change, it is now not uncommon to use sugar removing or de-alcoolization techniques because the grape berry sugar content becomes too high. A comprehensive understanding of sugar and acid accumulation and metabolism is crucial both for the selection and cultivation of superior grapevine varieties and for the optimization of agricultural practices aimed at enhancing fruit quality.

The sensitivity of grape leaves photosynthesis to various environmental factors (water, light and temperature) has been extensively studied (Jackson and Lombard 1993; Kolb et al. 2001; Hendrickson et al. 2004; Roig-Oliver et al. 2020; Rafique et al. 2023). However, it has been shown that the main driver of sugar accumulation in grape berries lies in the unloading process rather than in the ability of source leaves to synthesize and export photosynthetic sugars (Li et al. 2021). It involves two pathways that are not mutually exclusive: symplastic and apoplastic (Ruan et al. 2001; Viola et al. 2001; Zhang et al. 2006; Nie et al. 2010; Braun et al. 2014; Ren et al. 2023). Symplastic unloading through the plasmodesmata predominate during the early and mid-stages of grape berry development, while apoplastic unloading through the membranes becomes prominent at véraison (Zhang et al. 2022; Zhou et al. 2023). The plasmodesmata play a significant role in the switch from symplastic to apoplastic pathways (Zhang et al. 2006; Li et al. 2021; Zhou et al. 2023).

This switch is pivotal for the regulation of sugar accumulation (Zhang et al. 2006; Zhou et al. 2023). In addition to changes in plasmodesmatal density and permeability, it involves various enzymes of sugar metabolism, sugar transporter proteins and transcriptional regulators (Lecourieux et al. 2014; Durán-Soria et al. 2020; Li et al. 2021; Zenoni et al. 2023). Although this topic has been revised several times, significant and recent progress makes it useful to update it (Zhou et al. 2023; Liang et al. 2023). The present review highlights the challenges faced and future prospects, aiming to provide reference for in-depth studies into carbon accumulation in grape berries and thereby accelerate the breeding of high-quality grapes.

### Grape berry development and anatomical structure

The development and ripening of grape berries are commonly divided into three stages corresponding to different balances in sugars, acids, and phenolic compounds. The initial growth Stage I after fruit set is characterized by rapid cell division and expansion, resulting in an increase in berry size. During this stage, the berries are hard and green, with high acid and low sugar content (Harris et al. 1968; Kuhn et al. 2014). Stage II is a lag phase, characterized by slow growth. At the end of this stage, major physiological changes lead to véraison (onset of ripening) is visually marked by a change in berry color. From this time on, Stage III (ripening) is characterized by a massive hexose accumulation of hexoses, berry softening, and synthesis of secondary metabolites, including aromatic compounds (Coombe 1992; Lu et al. 2023).

**Fig. 1:**
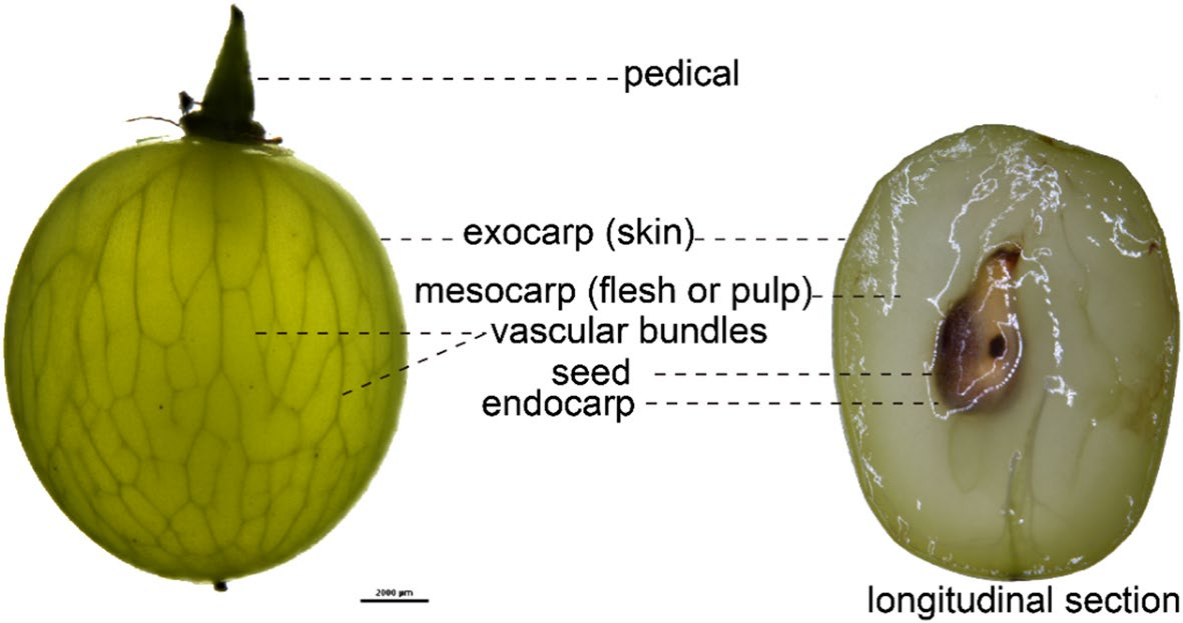
The anatomical structure of grape berry

Each berry is composed of the exocarp which provides color and contains aromatic compounds and tannins, the mesocarp which is the main reservoir of sugars and acids, the seeds which contribute tannins and oils, and the endocarp as the thin layer that delineates the boundary of the locular cavities where the seeds develop (Fig. 1) (Coombe 1987; Conde et al. 2007; Fontes et al. 2011; Candar 2023). The vascular system interconnects these parts, ensuring the transport of the compounds that allow fruit development (Zhang et al. 2006). During the three developmental stages, the berries undergo major biochemical transformations, including sucrose-hexoses conversion, a change in the acid/sugar balance, the synthesis of tannins and aromatic compounds, which collectively contribute to their sensory attributes (Conde et al. 2007; Martínez-Esteso et al. 2011; Kuhn et al. 2014; Perotti et al. 2023).

### Sugar and organic metabolism in grape berries

Grape berry metabolism involves a highly orchestrated interplay between sugar and acid biosynthesis, heavily reliant on photosynthetic carbon sources from the leaves (Sweetman et al. 2009). Sucrose, the main photoassimilate synthesized in the leaves, is translocated to the berries, forming the backbone for the synthesis of sugars and acids. The metabolism of sugars and organic acids undergo dramatic shifts at the véraison stage (Brady 1987; Giovannoni 2001, 2004; Maria et al. 2011; Giovannoni et al. 2017; Liu et al. 2023).

Before véraison, the berry engages in cell division and growth, accumulating organic acids, primarily malic acid, while sugar concentration remains at a low level (Conde et al. 2007; Dai et al. 2013; Etienne et al. 2013; Batista-Silva et al. 2018). At this stage, Sucrose is actively unloaded to berries and subsequently hydrolyzed by cell wall invertases (CWINV) into glucose and fructose (Maria et al. 2011; Kuhn et al. 2014,). After uptake by the flesh cells, glucose is further metabolized to phosphoenolpyruvate (PEP) by glycolysis. PEP lies at a critical crossroad leading to two separate pathways towards malate synthesis (Sweetman et al. 2009). PEP carboxylase (PEPC) catalyzes the conversion of PEP to oxaloacetate (OAA), which is then reduced to malate by NAD-dependent malate dehydrogenase (NAD-MDH) in the cytoplasm (Givan 1999). Alternatively, PEP may be converted by pyruvate kinase (PK) to form pyruvate, which can be further reduced to malate by NADP-dependent malic enzyme (NADP-ME) (Farineau and Lavalmartin 1977; Taureilles-Saurel et al. 1995; Sweetman et al. 2009; Martínez-Esteso et al. 2011). Then the malate can be transported into the mitochondrial matrix by malate transporter embedded in the inner mitochondrial membrane. Once inside, a mitochondrial NAD-dependent malate dehydrogenase converts malate to OAA and NADH, or a NAD-dependent malic enzyme converts it to pyruvate, CO_2_, and NADH (Sweetman et al. 2009). These intermediates feed the tricarboxylic acid (TCA) cycle, with the potential for malate regeneration depending on the metabolic flux within the mitochondria (Beriashvili and Beriashvili 1996; Ollat and Gaudillère 1997; Hanning et al. 1999). Excess malate is ultimately transported into the vacuoles, a process critical for maintaining the cytosolic pH balance and regulating the acid taste of the berry (Martínez-Esteso et al. 2011).

Grape berries exhibit a remarkable ability to synthesize and accumulate malate at pre-véraison stage, not only through the import of photosynthetically fixed carbon from the leaves, but also through the photosynthetic activity of exocarp cells (Sweetman et al. 2009; Garrido et al. 2023). Despite the limited presence of stomata in the berry skin, respiratory CO_2_ contributes to the synthesis of malate in flesh cells. Respiratory CO_2_ is converted to bicarbonate ion (HCO_3_^−^) by carbonic anhydrase within the cytoplasm (Blanke and Lenz 1989; Garrido et al. 2023,). Phosphoenolpyruvate carboxylase (PEPC) then catalyzes the formation of oxaloacetate (OAA) from HCO_3_^−^ and the formation of phosphoenolpyruvate (PEP) in an irreversible β-carboxylation reaction (Beriashvili and Beriashvili 1996; Sweetman et al. 2009). The OAA is subsequently reduced by NAD-MDH to form malate. The malate is not a metabolic end point; it can be shuttled into chloroplasts where it undergoes decarboxylation by NADP-ME (Maria et al. 2011; Garrido et al. 2023). This reaction releases CO_2_ which can be re-assimilated by ribulose-1,5-bisphosphate carboxylase/oxygenase (Rubisco) in the Calvin-Benson-Bassham (CBB) cycle (Conde et al. 2007). The pyruvate resulting from this decarboxylation can be converted back to PEP by pyruvate, phosphate dikinase (PPDK), resulting in a regenerative loop within carbon metabolism (Ruffner 1982; Sweetman et al. 2009; Etienne et al. 2013; Garrido et al. 2023). The interconversion of pyruvate and malate provides connectivity to other essential metabolic pathways (Garrido et al. 2021). Both pyruvate and malate can feed the tricarboxylic acid (TCA) cycle, supporting cellular respiration and biosynthetic reactions (Fig. 3) (Etienne et al. 2013). Alternatively, malate can accumulate in the vacuole, contributing to the grape’s acidity, or it can serve as a substrate for gluconeogenesis, influencing sugar concentrations (Dai et al. 2013; Etienne et al. 2013; Reshef et al. 2022). Moreover, potassium influences the pH and acidity of grape must, with higher potassium levels often associated with lower acidity due to the interaction with malate in the berries (Rogiers et al. 2017).

**Fig. 2:**
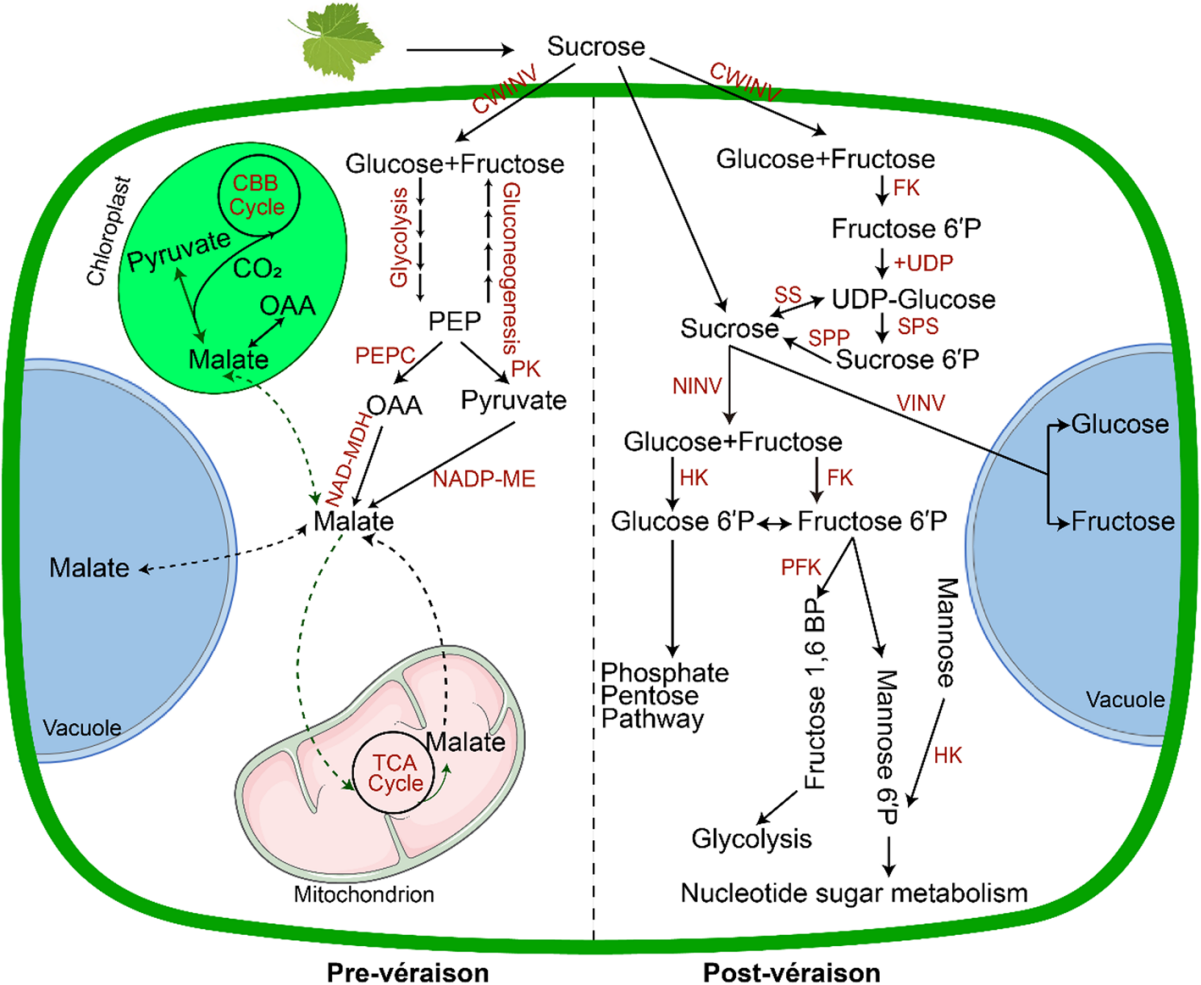
Sugar accumulation and sugar metabolism in the grape cells. PEP, phosphoenolpyruvate; OAA, oxaloacetic acid; CWINV, cell wall invertase; NINV, neutral invertase; VINV, vacuolar invertase; PEPC, phosphoenolpyruvate carboxylase; PK, pyruvate kinase; NAD-MDH, NAD-linked malic enzyme; NADP-ME, NADP-linked malic enzyme; FK, fructokinase; SS, sucrose synthase; SPS, sucrose phosphate synthase; SPP, sucrose phosphate phosphatase; HK, hexokinase; PFK, phosphofructokinase. CBB, Calvin-Benson-Bassham; TCA, tricarboxylic acid cycle

Post-véraison, there is an onset of hexose (glucose and fructose) accumulation and a concomitant decline in malate content (Davies and Robinson 1996). Sucrose metabolism is a central aspect of the biochemistry governing grape berry hexose accumulation (Ollat et al. 2002; Gambetta et al. 2010; Ruan 2014; Zhu et al. 2022). There is an overview of sugar metabolism in post-véraison berries (Fig. 2). At arrival in the berries, the sucrose transported by the phloem can be either hydrolyzed into glucose and fructose by invertases (INVs) or converted to UDPG and fructose by sucrose synthase (SS) (Li et al. 2012; Verma et al. 2011) (Fig. 2). Three types of invertases differ by their localization, cytosolic for the neutral invertase (NINV), vacuolar for the vacuolar invertase (VINV) and cell wall for the cell wall invertase (CWINV) (Ruan et al. 2010; Wang et al. 2014) (Fig. 2). The three types of invertase collectively ensure that hexose is available. SS provides an alternative route for sucrose degradation, generating fructose and UDP-glucose, which is particularly important for sustaining sucrose levels within cells (Verma et al. 2011). Hexokinase (HK) and fructokinase (FK) phosphorylate glucose and fructose to glucose-6-phosphate (G6P) and fructose-6-phosphate (F6P), respectively (Jang et al. 1997; Granot et al. 2013) (Fig. 2). Phosphofructokinase (PFK) then acts on F6P converting it to fructose-1,6-bisphosphate (F1,6BP), channeling it into glycolysis and subsequently into the TCA cycle, a key energy-producing pathway in respiration (Ronimus and Morgan 2001) (Fig. 2). Sucrose phosphate synthase (SPS) and sucrose phosphate phosphatase (SPP) cooperate in the resynthesis of sucrose, reutilizing the products of SS activity to regenerate sucrose from UDP-glucose and F6P (Huber and Huber 1996; Tian et al. 2012; Xia et al. 2021; Huang et al. 2022) (Fig. 2). This cycle, called “futile sucrose recycle” is not merely a metabolic detour but serves a regulatory function in balancing cellular energy and carbon partitioning, which is crucial during the stages of rapid growth and sugar accumulation of berries (Nguyen-Quoc and Foyer 2001).

**Fig. 3:**
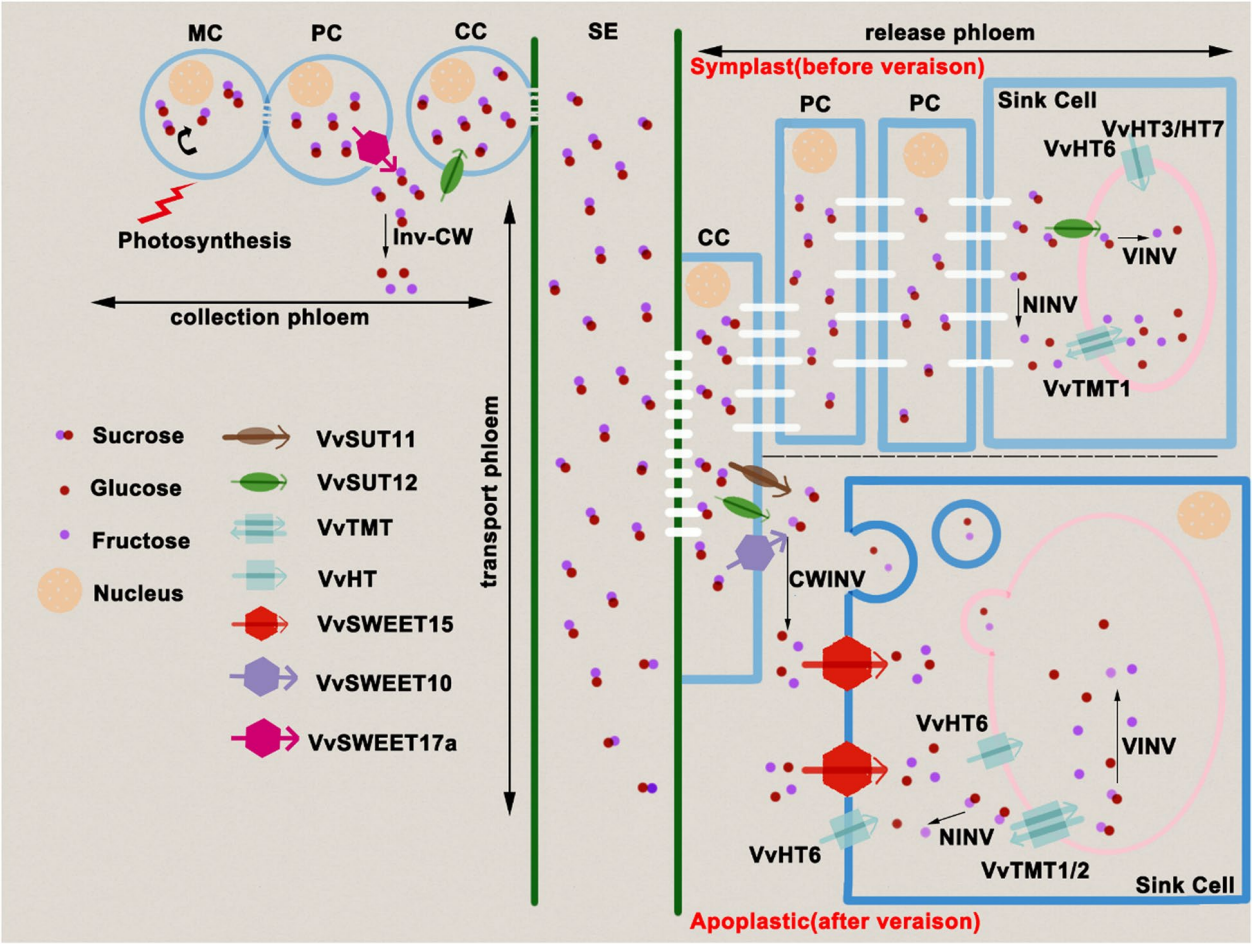
The hypothetical model of sugar transporters involved in sugar accumulation in the grape berries. MC, mesophyll cell; PC, parenchyma cell; CC, Companion cell; CBB, calvin-benson-bassham; CWINV/Inv-CW, cell wall invertase; VINV, vacuolar invertase; NINV, neutral invertase; SUT, sucrose transporter; SWEET, sugars will eventually be exported transporter; VvHT, hexose transporter; VvTMT, tonoplast monosaccharide transporter

The metabolism of sucrose in grape berries is a multifaceted process that involves several specialized enzymes operating in concert across different cellular locations. The coordinated activities of SUTs, INVs, and SS manage the distribution and conversion of sucrose, while the activities of HK, FK, PFK, SPS, and SPP ensure its utilization and recycling within the cellular environment. The ‘futile sucrose cycle’ plays a substantial role in the developmental process, ensuring the hexoses accumulation essential for fruit quality (Nguyen-Quoc and Foyer 2001). Understanding the interplay of these enzymes and their regulation factors provide critical insight for strategies aimed at optimizing sugar content in grape berries, which is paramount for achieving desired wine attributes. Future research aimed at quantitatively measuring these enzyme activities in vivo and identifying their regulatory mechanisms offers the prospect of fine-tuning grape berry composition in the context of ever-changing environmental challenges and winemaking goals.

### Sugar transporters in grape berries

Several families of sugar transporters play a key role in sugar accumulation during the ripening of grape berries. Therefore, these transporters are a key to understand the molecular mechanisms underlying these processes. The function of numerous sugar transport proteins has been elucidated (Reinders et al. 2012; Eom et al. 2015; Nino-Gonzalez et al. 2019; Wen et al. 2022; Pegler et al. 2023). Among these, three primary sugar transporter families have been identified as key contributors to sugar accumulation in plants: the Monosaccharide Transporters (MST), the Sucrose Transporters (SUT/SUC), and the Sugar Will Eventually be Exported Transporters (SWEET) protein families (Doidy et al. 2012; Pegler et al. 2023).

Members of the monosaccharide transporter (MST) family are ubiquitously distributed across plant species and are predicted to possess 12 transmembrane domains (Pao et al. 1998; Buttner and Sauer 2000; Buttner 2007). Within this family, seven subfamilies have been classified: Sugar Transport Proteins/Hexose Transporters (STP/HT), Tonoplast Sugar Transporters (TST, formerly known as TMT), Early Responsive to Dehydration Like 6 (ERDL6), Plastidic Glucose Transporters (pGlcT), Inositol Transporters (INT), Polyol/Monosaccharide Transporters (PMT, formerly known as PLT), and Vacuolar Glucose Transporters (VGT) (Buttner 2007; Slewinski 2011; Nino-Gonzalez et al. 2019). Notably, the STP/HT and TST subfamilies have been extensively studied.

MSTs have been acknowledged as pivotal in sugar accumulation (Fontes et al. 2011). In grapevine, a total of 59 monosaccharide transporter genes have been identified, which can be categorized into 7 subfamilies. It includes 20 VvHT (Subfamily I), 3 VvTMT (Subfamily II), 5 VvPMT (Subfamily III), 22 VvERDL6 (Subfamily IV), 2 VvVGT (Subfamily V), 3 VvINT (Subfamily VI), and 4 VvpGlcT/VvSGB1 (Subfamily VII) (Afoufa-Bastien et al. 2010). During berry development, the transcript levels of *VvHT3* and *VvHT6* are significantly higher than *VvHT1*, *VvHT2*, *VvHT4* and *VvHT5*. (Hayes et al. 2007; Afoufa-Bastien et al. 2010). The *VvHT1*, *VvHT2*, *VvHT4* and *VvHT5* are most lowly expressed through the grape berry development period (Hayes et al. 2007; Afoufa-Bastien et al. 2010). Notably, the expression of *VvHT3* is reduced at véraison but elevated highly in pre-véraison and post-véraison (Hayes et al. 2007). The expression of *VvHT1* is strong shortly after anthesis but decreased during the period of rapid sugar accumulation (Hayes et al. 2007). *VvHT6* expression remained high throughout the ripening process (Afoufa-Bastien et al. 2010). Immunofluorescence, immunolabeling and GFP fusion protein experiments revealed the plasma membrane localization of VvHT1, VvHT4, and VvHT5. VvHT1 exhibited higher glucose affinity and broader substrate specificity than VvHT4 and VvHT5, recognizing both D-glucose and D-fructose. VvHT3 was not capable of importing any sugar in mutant yeast strains (Vignault et al. 2005; Conde et al. 2006; Hayes et al. 2007). VvHT2 and VvHT6/VvTMT2 appear to be localized to the tonoplast, with *VvHT6*/*VvTMT2* showing high sequence similarity to *AtTMT2* (Agasse et al. 2009; Afoufa-Bastien et al. 2010). *VvTMT1* and *VvTMT2* exhibit higher expression levels in berries (Afoufa-Bastien et al. 2010). *VvTMT2* is notably high expressed at the onset of ripening and post-véraison stages in *V. vinifera* ‘Sultanine’ berries (Cakir et al. 2012). The fusion expression of VvTMT1-GFP in yeast demonstrated tonoplast localization, and VvTMT1 glucose uptake was heterologously assessed by yeast hexose transporter mutants (Zeng et al. 2011). The various localization and affinity for substrate among these monosaccharide transporters suggest that their functions of sugar transport are diverse.

Reinders reported that AtSUTs can be divided into three types: Type I, which includes AtSUC1, 2, 5, 6, 7, 8, and 9; Type II includes AtSUC3; Type III includes AtSUC4 (Reinders et al. 2012; Wen et al. 2022). SUT/SUC transporters primarily transport sucrose into the SE-CC complex (Scofield et al. 2007; Slewinski et al. 2009, 2010; Wang et al. 2021). Expression of *AtSUC2* can enhance sucrose loading in rice, thereby resulting in larger grains and improved crop yield (Wang et al. 2015). Suppressing tomato *SUT1* (Hackel et al. 2006), knocking out rice *SUT1* (Wang et al. 2021), and expressing *SUT1* in pea (Lu et al. 2020), have indicated that SUT/SUC class transporters are crucial for phloem loading. Arabidopsis AtSUC5 enables sucrose inflow into the endosperm, ultimately providing nutrition to the embryo (Baud et al. 2005). In seeds, OsSUT1/3/4, localized to the starchy layer, can transport sucrose into seeds to enhance sucrose unloading (Furbank et al. 2001; Bai et al. 2016). Sugarcane *SUT5* and *SUT6* are highly expressed in source leaves, aiding phloem loading (Zhang et al. 2016), SUT1 does not participate in phloem unloading but is involved in recycling sucrose leaked into the apoplast back to the vascular parenchyma cells (Glassop et al. 2017). Maize SUC4 is localized to the tonoplast and can export sucrose from vacuoles (Carpaneto et al. 2010; Schneider et al. 2012). Furthermore, AtSUC5 can also transport biotin (Ludwig et al. 2000), and AtSUC9 is able to transport a wide range of glucosides (Sivitz et al. 2007).

VvSUTs (VvSUC2, VvSUC11, VvSUC12, and VvSUC27) in different Vitis varieties focus on the expression, localization, function and regulation. *VvSUC2* exhibits low expression levels or not detected across various tissues and organs. *VvSUC27* is ubiquitously expressed in vegetative organs while is weakly expressed in berries (Afoufa-Bastien et al. 2010). The expression of *VvSUC11* and *VvSUC12* are relatively low in berries but stays stable during the ripening stages (Afoufa-Bastien et al. 2010). *VvSUC12* and *VvSUC27* were also expressed in seeds but at a lower level (Afoufa-Bastien et al. 2010). VvSUC11 and VvSUC12 with high-affinity/low-capacity to sucrose, control sugar distribution. VvSUC11, VvSUC12, and VvSUC27 can form homodimers and heterooligomers to guide the rapid transport of sucrose in SE (Cai et al. 2021). VvSUC27 is localized on the plasma membrane. Overexpressing *VvSUCs* (*VvSUC11* or *VvSUC12* or *VvSUC27*) in tobacco and Arabidopsis showed that the plants grew faster, had increased yield, and enhanced stress resistance (Cai et al. 2017, 2020). Similarly, SUTs in grape “Zuoshan-1” responded to various stresses, promoting sucrose metabolism and hormone synthesis (Cai et al. 2019). However, the research of VvSUTs function is still predominantly conducted in heterologous systems, such as Arabidopsis, tobacco. In fact, a direct assessment of their roles in sugar accumulation in grape berries is limited or almost non-existent. This gap highlights the need for more research in grape berries to fully understand the contributions of VvSUTs in sugar accumulation and ripening.

SWEETs are a novel transporter family in plants involved in cellular sugar efflux (Chen et al. 2010), primarily transporting substrates such as glucose, fructose, and sucrose (Chardon et al. 2013; Klemens et al. 2013; Eom et al. 2015). In angiosperms, there are an average of 20 SWEET family members, which are differentially expressed across diverse tissues and organs. In Arabidopsis, SWEET members are phylogenetically divided into four clades, with Clade I (SWEET1-2), Clade II (SWEET3-8), and Clade IV (SWEET16-17) mainly transporting monosaccharides, whereas Clade III (SWEET9-15) mainly transports sucrose (Chen et al. 2010, 2015). SWEET transporters can be localized in various subcellular compartments: SWEET1, 8, 9, 11, 12, and 15 are primarily localized to the plasma membrane (Seo et al. 2011; Kryvoruchko et al. 2016), SWEET2, 16, and 17 to the tonoplast (Chardon et al. 2013; Klemens et al. 2013; Guo et al. 2014; Chen et al. 2015), and SWEET9 to the Golgi membrane (Lin et al. 2014; Chen et al. 2015). SWEET proteins are involved in various functions including plant carbon partitioning, pollen nutrition supply, seed development, organ senescence, hormone transport and interactions between plants and pathogens (Chen et al. 2015; Hutin et al. 2015; Ho et al. 2019; Ni et al. 2020; Braun 2022; Xue et al. 2022; Radchuk et al. 2023). As research continues to deepen, the regulatory networks of SWEET proteins and their potential in improving crop yield and stress resistance are expected to be more comprehensively assessed and utilized.

In grapevine, there are 17 *SWEET* homologues, among which among which *VvSWEETs* (*VvSWEET1*, *VvSWEET2a*, *VvSWEET2b*, *VvSWEET4*, *VvSWEET7*, *VvSWEET10*, *VvSWEET15* and *VvSWEET17a*) have been identified as being expressed during grape berries development. Among them, *VvSWEET1*, *VvSWEET2a*, *VvSWEET2b*, *VvSWEET10*, *VvSWEET15*, and *VvSWEET17a* displayed higher expression in Chardonnay berries than those in other organs (Zhang et al. 2019). *VvSWEET10* is highly expressed in véraison (Zhang et al. 2019). Specifically, *VvSWEET15* is strongly expressed in both véraison and post-véraison in Chardonnay berries and the expression level is much higher than that of *VvSWEETs* (*VvSWEET1*, *VvSWEET2a*, *VvSWEET2b*, *VvSWEET10*, *VvSWEET15* and *VvSWEET17a*) (Zhang et al. 2019). VvSWEET10, a plasma membrane transporter, was found to significantly increase glucose, fructose, and total sugar content when overexpressed in grape callus and tomato (Zhang et al. 2019). *VvSWEET15* was highly expressed in the three grape varieties and was positively correlated with the hexose content during ripening (Ren et al. 2020). In our research, *VvSWEET10* and *VvSWEET15* exhibit notably high expression level in grape berry and an in-depth gene-silencing and overexpressing studies of *VvSWEET15* demonstrate that VvSWEET15 facilitates hexose accumulation at post-véraison stages (unpublished data). Future research is required to explore these potential roles and deepen understanding of the molecular mechanisms underlying grape ripening and sweetness. *VvSWEET4* is lowly expressed in small green berry and pre-version green berry, but is highly expressed in post-version berry of *V. vinifera* 40,024 (Chong et al. 2014). VvSWEET4 is a glucose transporter located on the plasma membrane (Chong et al. 2014). Overexpression of *VvSWEET4* in grapevine root hairs led to increased glucose content in the root hairs, upregulation of genes in the flavonoid biosynthetic pathway, and enhanced resistance to soil pathogen infection (Meteier et al. 2019). *VvSWEET7* is highly expressed during both the green berry phase and ripening phase in Trincadeira grapes and is able to transport monosaccharides, disaccharides, and polyols (Breia et al. 2020). VvSWEET7 may participate in plant defense by rapidly removing pathogen-synthesized mannitol from the extracellular space (Breia et al. 2020). The expression of *VvSWEET2a*, *VvSWEET7*, and *VvSWEET15* increases significantly when grapes are infected by Botrytis cinerea, whereas different developmental stages of infection downregulate the expression of *VvSWEET10*, *11*, *17a*, and *17d* (Breia et al. 2020).

Sugar transporters present a complex, critical network essential for sugar accumulation in grape berries. The precise expression patterns of these transporters—coordinated with development stages, specificity for sugar substrates, and cellular localization—reflect the intricate control of sugar distribution within the berry. The current understanding of sugar transport proteins in grape berries is limited due to restrictions in transgenic systems, leaving their exact functions somewhat unclear. However, to enhance our knowledge of the role of sugar transport proteins in sugar accumulation in grape berries, we have objectively organized the existing data into a hypothetical model (Fig. 3). Given that *VvSUC12* has two structural features unique to the *SUT2/SUC3* subfamily (including *AtSUC3*), which shares a 66.6% similarity with *AtSUT3* and is expressed in mature grape leaves, it is hypothesized to be involved in loading sucrose into the phloem SE-CC complex, akin to AtSUT3’s function in sucrose funneling from the mesophyll towards the phloem (Meyer et al. 2000; Afoufa-Bastien et al. 2010) (Fig. 3). *VvSWEET17a*, with high expression in mature leaves, might function similarly to AtSWEET11 and 12, facilitating sucrose across the plasma membrane from mesophyll cells to the apoplastic space (Chen et al. 2012) (Fig. 3). This suggests that SWEET17a carries sucrose across the plasma membrane and VvSUT12 further move it into the phloem.

Subsequently, sucrose is transported long distances through the phloem and eventually reaches the unloading phloem. Here, it is speculated that sucrose is released into the apoplastic space through VvSUT11, VvSUT12, and VvSWEET10 (Fig. 3). The functions of VvSUT11 and VvSUT12 are similar to the sucrose efflux functions of homologs AtSUT4 (belongs to the SUT4 subfamily, including AtSUC4) and AtSUT3 respectively (Manning et al. 2001; Afoufa-Bastien et al. 2010). The function of SWEET10 in sucrose transport has been demonstrated in sucrose-deficient yeast (unpublished data from our laboratory) (Fig. 3). Most studies suggest that released sucrose is mainly translocated through symplastic transport via plasmodesmata and eventually accumulates hexoses via plasma membrane-located HTs, as well as vacuolar membrane transporters-located TMTs and HTs at pre-véraison (Breia et al. 2021; Braun et al. 2022; Wen et al. 2022; Ren et al. 2023). At this stage, the HTs are hypothesized to be VvHT3/VvHT7 and VvHT6, the TMTs are VvTMT1 and VvTMT2, according to their transcriptome data (Afoufa-Bastien et al. 2010; unpublished expressing data from our laboratory) (Fig. 3). After véraison, part of the released sucrose is degraded into hexoses by cell wall invertase (CWINV) and neutral invertase (NINV), which are then transported across the plasma membrane by VvHT6 and VvSWEET15, and across the vacuolar membrane by VvTMT1, VvTMT2, VvHT6, and VvSWEET15, ultimately accumulating hexoses in the vacuole (Hayes et al. 2007; Afoufa-Bastien et al. 2010; unpublished expressing data from our laboratory) (Fig. 3). This narrative reflects a hypothesis based on the current limited data and requires further research for confirmation.

Overall, the development of grape berries is characterized by the continuous accumulation of sugars, which forms many important carbohydrates in mature berries and ultimately dictates berry yield and quality (Smeekens 2000; Rolland et al. 2002). In grapevine, sugar is exported from source leaves and eventually accumulates in the FCs through symplastic and apoplastic pathways. Sucrose is synthesized in the photosynthetic mesophyll cells of leaves and loaded into phloem sieve tubes via symplastic or apoplastic loading pathways, which is propelled by hydrostatic pressure. After long-distance transport, sucrose is unloaded at the site of CCs and transported into the apoplastic space, subsequently either transported by VvSUTs on the plasma of PCs or cleaved into hexoses by CWINV or SS (Chen et al. 2017; Wan et al. 2018; Duan et al. 2020). These hexoses are later transported into the FCs through specific VvPMTs located on the plasma or vacuolar membranes (Grappadelli et al. 2019). These processes maintain a sucrose concentration gradient at unloading sites, ensuring rapid unloading and accumulation of hexose (Lecourieux et al. 2014). Research indicates that the phloem unloading pathway in berries undergoes a transition during berry development, shifting from symplastic to apoplastic unloading pathway around the time of véraison (Zhang et al. 2006). As grape berries develop into the post-véraison stage, the deposition of callose blocks plasmodesmata, resulting in the symplastic isolation of between CCs and PCs/FCs (Zhang et al. 2006). This isolation only allows sucrose to be unloaded through VvSUTs on the CC plasma membranes into the apoplastic space, where hexose ultimately transported into the FCs via apoplastic unloading pathways. Thus, sugar transporters and enzymes associated with sugar metabolism together form a complex regulatory network that governs sugar accumulation in grape berries. Understanding these intricate interactions between sugar transporters and various metabolic pathways enables researchers to devise strategies for manipulating sugar distribution and metabolism, which can enhance fruit quality and prolong shelf life.

### Regulatory factors influencing sugar accumulation

Despite extensive research on the function of sugar transporters in various plant species, reports focusing on the transcriptional and post-translational regulation of these proteins are relatively scarce. The current knowledge predominantly addresses transcriptional regulation of a limited number of transporter genes and less is known about the post-translational control mechanisms that modulate transporter activity.

Sugar transporters at transcription level only concentrated on minority. For instance, in grapevine, the interaction between *VvMSA* and the *VvHT1* promoter indicates a positive regulation of *VvHT1* promoter activity (Çakir et al. 2003). The R2R3-type MYB96 transcription factor directly binds to the *STP13* promoter activating its expression, which induces sugar uptake and enhances plant tolerance to adverse environmental challenges (Lee and Seo 2021). In watermelon, the transcription factor SUSIWM1 positively regulates *ClTST2* gene, promoting the accumulation of sucrose, glucose, and fructose in the flesh cell vacuoles (Ren et al. 2018). In rice, NF-YB1 influences the expression of *OsSUT1/3/4* located in the aleurone layer, intensifying sucrose unloading (Furbank et al. 2001; Bai et al. 2016). The *OsDOF11* transcription factor binds to the promoter regions of *OsSUT1*, *OsSWEET11*, and *OsSWEET14* enhancing the expression of these genes, thereby affecting sugar transport in rice. The mutant *Osdof11* exhibits dwarfed stature, reduced tillering, insensitivity to sucrose-mediated root growth inhibition, reduced sugar accumulation in leaves, and diminished phloem sucrose flow. The ABA-responsive transcription factor OsbZIP72 can bind to the promoter regions of *OsSWEET13* and *OsSWEET15*, activating their expression in response to drought stress (Mathan et al. 2021). In cotton, the transcription factor GhMYB212 binds to the *GhSWEET12* promoter, promoting its expression to regulate the carbon supply required for cotton fiber elongation (Sun et al. 2019). Within pear fruit, PuWRKY31 directly binds to the *PuSWEET15* promoter, upregulating its expression and enhancing high sucrose accumulation in the fruit of high-sugar bud sports (Li et al. 2020). The lily transcription factor LoABF2 (an AREB/ABF binding factor) can bind to the *LoSWEET14* promoter, inducing *LoSWEET14* expression and participating in the ABA signaling pathway to promote soluble sugar accumulation in response to various abiotic stresses (Zeng et al. 2022). The VvMYB15 transcription factor is implicated activating the expression of *VvSWET15* (Li et al. 2022). In apple (Malus × domestica) variety “Gala”, MdWRKY9 which bound to the *MdSWEET9b* promoter interacted with MdbZIP23 (basic leucine zipper) and MdbZIP46, and upregulated *MdSWEET9b* expression, thereby influenced apple fruit sugar accumulation (Zhang et al. 2023).

Post-translational research on sugar transporters primarily focuses on control by kinases and phosphatases. For example, the expression of monosaccharide transporters (*VvHT3*, *VvHT4*, *VvHT5*, and *VvHT6*) in grapevine is regulated by protein kinases (VvSK1), modulating sugar intake and accumulation (Lecourieux et al. 2010). Glucose can inhibit the transcription of *VvHT1* via a process dependent on hexokinase (HXK) and can reduce the abundance of VvHT1 protein in the plasma membrane through HXK-mediated post-translational modifications (Conde et al. 2006). In Arabidopsis, the wall-associated kinase AtWAKL8 acts as a positive regulator of AtSUC2, capable of phosphorylating AtSUC2 thereby enhancing its sucrose-binding capacity (Xu et al. 2020). The ethylene-responsive transcription factor MaRAP2-4 activates the expression of the Arabidopsis *SWEET10*, modulating sugar accumulation to increase waterlogging tolerance and enhance the drought and salt tolerance of the Lamiaceae species (*Mentha arvensis*) (Phukan et al. 2018). Additionally, the transport activity of sugar transporters can be regulated through interaction with binding proteins. In potato, the interaction between StSP6A and StSWEET11 prevents the leakage of sucrose into the apoplastic space during tuber development and leads to reduced transport activity of StSWEET11 when bound to StSP6A in protoplasts and yeast (Abelenda et al. 2019). Rice copper transporters (OsCOPT1 and OsCOPT5) interact with OsSWEET11 to modulate copper distribution during infection with Xoo, although it is not yet clear if this interaction affects the sugar transport of OsSWEET11 (Yuan et al. 2010).

The transcriptional and post-translational regulation of sugar transporters uncover a complex network dictating the functional state of these proteins. Transcription factors orchestrate the transcriptional response to developmental cues and environmental stimuli, while kinases and phosphatases finely tune transporter activity to adapt to cellular needs. As research progresses, elucidating the precise dynamic regulatory mechanisms will be crucial for a more comprehensive understanding of sugar transport in plants, especially in grapevine, with implications for agricultural productivity and stress resilience.

### Environmental factors influencing sugar accumulation

Temperature poses significant threats to viticulture in current and future global climate change scenarios (Venios et al. 2020). Temperature significantly influences grapevine metabolism and consequently sugar accumulation in grapes. Warmer temperatures accelerate the rate of sugar accumulation (measured in Brix) by enhancing photosynthetic activity in leaves, which leads to increased sugar production and transport to the berries (Stanfield et al. 2024). However, the highest quality wine is produced when the berries simultaneously achieve optimal sugar-to-acid ratios and maximum levels of pigments, aromas, and flavors (Gladstones 2011). High temperatures accelerate sugar accumulation in grape berries, leading growers to harvest early to avoid producing overly sweet, flat-tasting wines with high alcohol content, although the berries have not yet reached optimal flavor development (Delrot et al. 2020). This creates a challenge for winemakers because the sugars and flavors contents develop at different rates. To address this issue, growers select grape cultivars from hotter wine regions that possess traits enhancing hydraulic resistance. This adaptation helps improve wine quality by slowing the rate of sugar accumulation (Stanfield et al. 2024).

Sunlight exposure plays a pivotal role in shaping the quality of grape bunches and berries, significantly affecting the physiological and metabolic pathways of grapevines and ultimately influencing sugar accumulation in grapes (Friedel et al. 2015). Increased sunlight exposure boosts photosynthesis rates, potentially enhancing sugar availability for berry development. Berries that are fully exposed to sunlight tend to have smaller diameters and higher total soluble solids (up to 22.4 Brix) with lower acidity and juice pH compared to those in partial or complete shade (Somkuwar et al. 2023). This exposure also increases levels of hydroxybenzoic acid, gallic acid, ellagic acid, and anthocyanins, while decreasing flavan-3-ols and amino acids compared to shaded berries (Downey et al. 2004; Somkuwar et al. 2023). In contrast, shaded bunches show higher proline concentrations, underlining the profound impact of sunlight on the biochemical composition and quality of grape berries (Moukarzel et al. 2023). Additionally, the temperature of berry skins, elevated by direct sunlight, affects enzymatic activities crucial for sugar metabolism. Sunlight also influences the expression of genes involved in sugar transport and metabolism, further impacting sugar accumulation (Moukarzel et al. 2023). However, excessive sunlight or heat can cause detrimental effects like berry sunburn and reduced photosynthetic efficiency, potentially diminishing sugar content of berries (Gambetta et al. 2021). Therefore, achieving optimal sunlight exposure through proper vineyard management practices such as leaf removal, shoot positioning, and vine spacing is essential for maximizing sugar content and enhancing grape quality, which are vital for the final quality of wine (Smart 1985; Palliotti et al. 2011; Reynolds 2022).

### Genetic diversity of sugar accumulation in grape berries

Within the *Vitis* genus, there is considerable genetic variability in both sugar composition and concentration. the total sugar concentration, commonly quantified as total soluble solids (TSS), ranges from 18.7 to 27 Brix at maturity across 78 cultivars of *Vitis vinifera*, which includes both table grape and wine grape varieties (Kliewer 1967a). Kliewer found a broader variation among 26 *Vitis* species from North America and the Middle East, with TSS at maturity spanning from 13.7 Brix in V. champinii to 31.5 Brix in V. riparia from Wyoming (Kliewer 1967b). Furthermore, among 18 Eurasian grape species in Xinjiang region, the TSS at maturity have been reported to vary widely, from as low as14.9 Brix in Victoria to as high as 25.1 Brix in Summer Black (Zhong et al. 2023). In terms of specific sugar types, all cultivars primarily accumulate glucose and fructose, typically glucose content ranging from 42.13 to 46.80% of the total sugar and the fructose contents varied from 42.68 to 50.95%, while exhibiting very low levels of sucrose which varied from 6.17 to 12.69% (Zhong et al. 2023). Conversely, *V. labrusca* and *V. rotundifolia*, along with their interspecific hybrids, are noted for higher sucrose levels, ranging from 5 to 58.28 g/L, along with moderate concentrations of glucose and fructose (from 35 to 54 g/L), marking a distinct contrast in sugar profiles (Dai et al. 2011).

Cultivated *V. vinifera* has a significantly higher concentration of soluble sugars compared to wild *Vitis* species. To explore how gene variations related to sugar metabolism and transport contribute to the higher sugar accumulation in cultivated *V. vinifera* compared to wild *Vitis* species, the genomes of 14 *V. vinifera* and 13 wild species were resequenced (Xin et al. 2013). Eleven gene families pertinent to sugar metabolism, identified from the *V. vinifera* 12 X genome, included two families involved in sucrose synthesis: SPS and SPP. SPS synthesizes sucrose 6-phosphate using UDP-glucose and D-fructose 6-phosphate, which SPP then converts into sucrose. This sucrose is further processed into UDP-glucose and fructose by sucrose synthase (SUSy), and into glucose and fructose by invertase (INV). Invertases, categorized into three subfamilies based on their biochemical properties and subcellular locations, play key roles in these conversions (Sturm 1999; Nonis et al. 2008). Additionally, seven enzymes associated with glycolysis were identified, including FK, HK, and others (Xin et al. 2013). Alongside sugar transporter genes reported in grapes, 138 DNA regions on the *V. vinifera* genome were examined to assess the impact of domestication on sugar content in grapes (Afoufa-Bastien et al. 2010; Xin et al. 2013).

Rapid progress in DNA sequencing and genotyping has enabled more effective Whole Genome Amplification (WGA) studies, especially in species with sparse genetic data (Lijavetzky et al. 2007; Pindo et al. 2008; Xia et al. 2009; Lam et al. 2010; Dong et al. 2023). This has led to a significant increase in the identification of genetic variations like SNPs and InDels, which are crucial for understanding genetic diversity and relationships across different accessions (Myles et al. 2011). A recent study analyzed the genetic diversity of grapevine by resequencing genomic DNA from 27 *V. vinifera* and wild *Vitis* species, producing 46.9 Gb of DNA sequences (Xin et al. 2013). Despite a low alignment rate with the reference genome, possibly due to its incompleteness or the substantial genetic variation between the samples and the reference, the researchers identified thousands of SNPs and InDels that suggest significant genetic diversity and divergence due to domestication (Xin et al. 2013). They discovered genes involved in sugar metabolism that exhibited considerable differences in SNPs/InDels between wild and cultivated grapes, underscoring the role of these genes in grape berry development and sugar accumulation (Xin et al. 2013). This genetic exploration not only enhances our understanding of influence of artificial selection on grapevine genetics biological mechanisms underlying sugar accumulation but also provided insights into the evolutionary dynamics that continue to shape this species.

## Conclusion

The journey from flowering to the harvest of sweet ripe grape berries results depends on the supply of sugars, on a complex interplay between acid and sugar metabolism, the efficiency of sugar transport systems, and regulatory factors orchestrating these processes (Lucas et al. 2013; Griesser et al. 2024). While considerable progress has unraveled various sugar metabolism pathways and function of the enzymes, the roles and regulation of sugar transport proteins (SUC, HT, TMT, SWEET) in diverse fruit crops, their cellular localization, and the exact operational dynamics of these proteins within fruit tissues largely remain elusive (Lecourieux et al. 2014; Li et al. 2021; Ren et al. 2023). Enhanced knowledge on these fronts bears the promise of paving the way for advancing grapevine cultivation, enology, and viticultural practices.

## Data Availability

Not applicable.

## Abbreviations

CBB: Calvin-benson-bassham
CWINV: Cell wall invertase
ERDL6: Early responsive to dehydration like 6
FK: Fructokinase
HK: Hexokinase
INT: Inositol transporters
INV: Invertase
MST: Monosaccharide transporter
NINV: Neutral invertase
NAD-MDH: NAD-linked malic enzyme
NADP-ME: NADP-linked malic enzyme
OAA: Oxalacetic acid
PEP: Phosphoenolpyruvate
PEPC: Phosphoenolpyruvate carboxylase
PFK: Phosphofructokinase
pGlcT: Plastidic glucose transporter
PK: Pyruvate kinase
PMT: Polyol monosaccharide transporter
SS: Sucrose synthase
SPS: Sucrose phosphate synthase
SPP: Sucrose phosphate phosphatase
STP: Sugar transport protein
SUT/SUC: Sucrose transporter
SUSy: Sucrose synthase
SWEET: Sugars will eventually be exported transporter
TCA: Tricarboxylic acid cycle
TST: Tonoplast sugar transporter
VGT: Vacuolar glucose transporter
VINV: Vacuolar invertase
VvHT: Hexose transporter
VvTMT: Tonoplast monosaccharide transporter
VvPMT: Polyol/monosaccharide transporter
VvVGT: Vacuolar glucose transporter
VvINT: Inositol transporter
WGA: Whole Genome Amplification
